# Aortic acceleration as a noninvasive index of left ventricular contractility in the mouse

**DOI:** 10.1038/s41598-020-79866-y

**Published:** 2021-01-12

**Authors:** Jorge Enrique Tovar Perez, Jesus Ortiz-Urbina, Celia Pena Heredia, Thuy T. Pham, Sridhar Madala, Craig J. Hartley, Mark L. Entman, George E. Taffet, Anilkumar K. Reddy

**Affiliations:** 1 Section of Cardiovascular Research, Department of Medicine, Baylor College of Medicine, One Baylor Plaza, MS:BCM285, Houston, TX 77030 USA; 2 Houston Methodist Hospital, Houston, TX USA; 3 Texas A&M University, Houston, TX USA; 4 Indus Instruments, Webster, TX USA

**Keywords:** Cardiovascular models, Ultrasound

## Abstract

The maximum value of the first derivative of the invasively measured left ventricular (LV) pressure (+ dP/dt_max_ or P′) is often used to quantify LV contractility, which in mice is limited to a single terminal study. Thus, determination of P′ in mouse longitudinal/serial studies requires a group of mice at each desired time point resulting in “pseudo” serial measurements. Alternatively, a noninvasive surrogate for P′ will allow for repeated measurements on the same group of mice, thereby minimizing physiological variability and requiring fewer animals. In this study we evaluated aortic acceleration and other parameters of aortic flow velocity as noninvasive indices of LV contractility in mice. We simultaneously measured LV pressure invasively with an intravascular pressure catheter and aortic flow velocity noninvasively with a pulsed Doppler probe in mice, at baseline and after the administration of the positive inotrope, dobutamine. Regression analysis of P′ versus peak aortic velocity (v_p_), peak velocity squared/rise time (v_p_^2^/T), peak (+ dv_p_/dt or v′_p_) and mean (+ dv_m_/dt or v′_m_) aortic acceleration showed a high degree of association (P′ versus: v_p_, r^2^ = 0.77; v_p_^2^/T, r^2^ = 0.86; v′_p_, r^2^ = 0.80; and v′_m_, r^2^ = 0.89). The results suggest that mean or peak aortic acceleration or the other parameters may be used as a noninvasive index of LV contractility.

## Introduction

The maximum value of the first derivative of left ventricular (LV) pressure (+ dP/dt_max_ or P′) is often used to quantify ventricular contractility. It is derived from the high-fidelity pressure signal measured invasively, using an intravascular pressure catheter. The invasive technique is frequently used in patients^1,2^ and in large animal research^3,4^. While complications are infrequent, repeated invasive pressure measurements are avoided in patients^5^. Repeated measurements of invasive pressure can be utilized in large animal research, but the invasive nature of this measurement has motivated investigators^5–12^ to develop noninvasive methods to measure ventricular contractility.

The shift in research from large to small animals such as mice and rats has compelled investigators to adapt techniques and methods designed for large animals for use in small animals^13^. While the measurement of ventricular pressure in mice was first reported in the 1930s^14^, its utility to measure contractility in mice began in the 1990s^15^ and many studies in small animals ensued thereafter^16–24^. However, invasive pressure measurement is problematic in rodents and other small animals as it is limited to a single or a few acute measurements and the study is terminal. The ability to measure LV contractility noninvasively allows researchers to pursue longitudinal studies using fewer animals.

Many indices of cardiac contractility have been developed in humans and large animals in the last several decades, which have been summarized in the report by Lambert et al.^25^ based on theoretical (references 9–17 of^25^), empirical (references 1–5 of^25^), and borrowed concepts (references 6–8 of^25^). The sensitivity of 24 of these (invasively & noninvasively derived) indices has been evaluated and ranked by Lambert et al.^25^ through their cardiac contractility study in dogs in the order from the most sensitive to the least sensitive. The first 7 most sensitive indices in their ranking require the invasively measured pressure signal. The next most sensitive indices of their ranking were the noninvasively measured mean and peak aortic accelerations, followed by the invasively measured first derivative of pressure (dP/dt). Therefore, we undertook this study to evaluate and validate aortic acceleration and other aortic parameters derived from Doppler aortic flow velocity as potential noninvasive surrogates to assess LV contractility in mice.

## Materials and methods

Studies were conducted in 10 wild-type mice (group 1: avg. age 5 months; 4 males and 6 females; C57BL/6J) and in 6 wild-type mice (group 2: avg. age 9 months, 3 males and 3 females; C57BL/6J). Mice were housed in the animal facility at the Neurosensory vivarium of Baylor College of Medicine. This facility is approved by the American Association for Accreditation of Laboratory Animal Care. Animals were kept in rooms at controlled temperature (24 °C) and lighting (14:10 h light–dark cycle) with free access to food and water. The diets of both groups of mice consisted of normal chow.

Mice from group 1 were anesthetized with 3% isoflurane in oxygen in the induction chamber and then transferred to a heated (38 ± 1 °C) electrocardiogram (ECG) board (Rodent Surgical Monitor, Indus Instruments, Webster, TX) with the limbs taped to electrodes and 1.5% isoflurane anesthesia delivered continuously via nose cone. Body fur was shaved in the neck area and prepared for right carotid artery cannulation. Mice from group 2 underwent similar preparation but body fur was shaved in the chest area to allow for simultaneous Doppler and echocardiography measurements. All animal protocols were approved by the Institutional Animal Care and Use Committee of Baylor College of Medicine in accordance with the National Institutes of Health Guide for the Care and Use of Laboratory Animals (National Research Council 2011. *Guide for the Care and Use of Laboratory Animals*: 8^th^ Ed. Washington, DC: The National Academies Press). The following experimental studies were conducted in the two groups of mice.

**Table:** 

Group 1: Ten mice	Group 2: Six mice
Simultaneous invasive pressure and noninvasive Doppler measurements	Day 1: Simultaneous noninvasive Doppler and echocardiography measurements Day 4: Noninvasive Doppler measurements Day 7: Noninvasive Doppler measurements

### Simultaneous invasive pressure and noninvasive Doppler measurements

**Figure 1:**
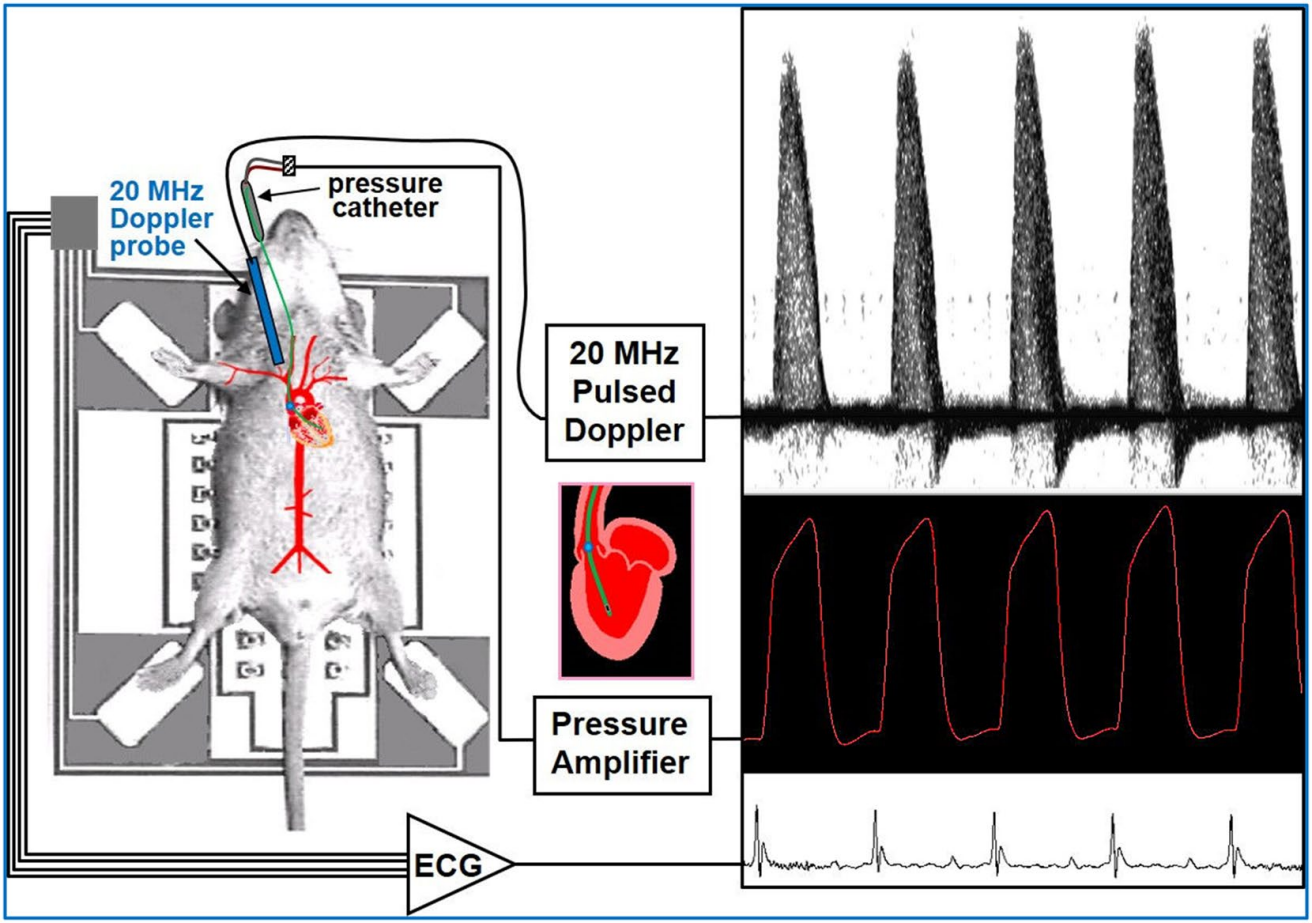
Experimental setup to simultaneously measure ascending aortic flow velocity using DFVS, left ventricular pressure using Millar catheter, and ECG in group 1 mice. Here the 20 MHz Doppler probe is oriented (angle ≤ 20°) towards the ascending aorta with sample volume placed at aortic root. Shown in the plot are ascending aortic outflow velocity (sampled at the site shown by blue dot), LV pressure (sampled at catheter tip in LV), and ECG signals.

**Figure 2:**
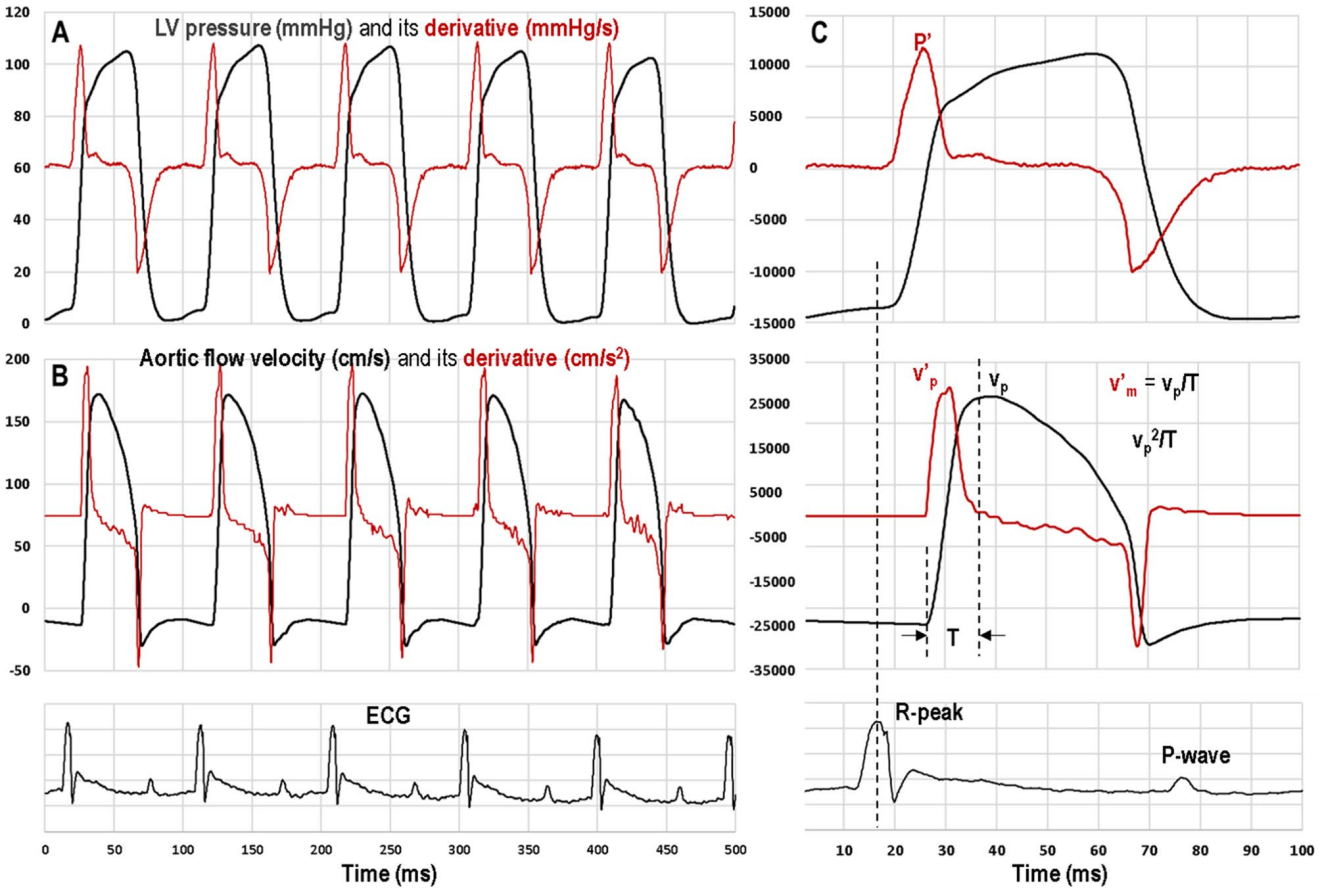
Waveforms of (**A**) invasively measured left ventricular pressure (P) waveform and its first derivative (P′) and (**B**) noninvasively measured aortic outflow velocity (v) waveform and its first derivative (v′). (**C**) Figure showing the invasive parameter left ventricular P′ and the noninvasive parameters peak aortic velocity (v_p_), peak aortic velocity squared/rise time (v_p_^2^/T), peak aortic acceleration (v′_p_) and mean aortic acceleration (v′_m_). Also shown is the ECG waveform and the time in milliseconds.

The invasive (aortic and left ventricular blood pressure) and noninvasive (ascending aorta blood flow velocity) measurements were made simultaneously. The surgical procedure to cannulate the mouse right carotid artery for intravascular and intraventricular pressure measurements was done as described previously^21^. Briefly, the catheter and pressure amplifier were calibrated for each experiment from 0 to 250 mm Hg using a mercury manometer. Blunt dissection techniques were used to expose the right carotid artery for cannulation with an intravascular pressure catheter (SPR1000 - Millar Mikro-tip catheter transducer, Millar, Inc., Houston, TX). The catheter was initially advanced into the ascending aorta and held in place. Aortic blood flow velocity was measured using a 20-MHz pulsed Doppler probe and a real-time signal acquisition and spectrum analyzer system (DFVS-Doppler Flow Velocity System, Indus Instruments, Houston, TX). The Doppler probe was placed slightly to the right of the suprasternal notch and oriented towards the heart and aortic root, the probe orientation adjusted with a micromanipulator in the direction from the right ear of the mouse towards the ascending aorta (Fig. 1). The probe was positioned such that the angle between the sound beam and the direction of aortic flow was less than 20° (velocity underestimation error ≤ 6.03%), and the range gate depth was adjusted between 3 and 4 mm to obtain maximal velocity. With the aortic pressure and velocity signals being displayed simultaneously, a 3-s segment of both signals was recorded. The catheter was then advanced into the LV. Again, 3-s segments of LV pressure and aortic flow velocity signals were recorded at baseline and after IP administration of single dose (1.5 μg/g) positive ionotropic agent dobutamine^21^. Maximal first derivative of LV pressure (+ dP/dt_max_ or P′) was calculated from the LV pressure signal. Aortic flow velocity waveforms were processed to calculate peak aortic velocity (v_p_), peak aortic velocity squared/rise time (v_p_^2^/T), peak (v′_p_) aortic acceleration, and mean (v′_m_) aortic acceleration. As shown in Fig. 2, v′_m_ is calculated as v_p_/T, where T is the rise time or the time from foot of the waveform to v_p_.

### Simultaneous noninvasive Doppler and echocardiography measurements

**Figure 3:**
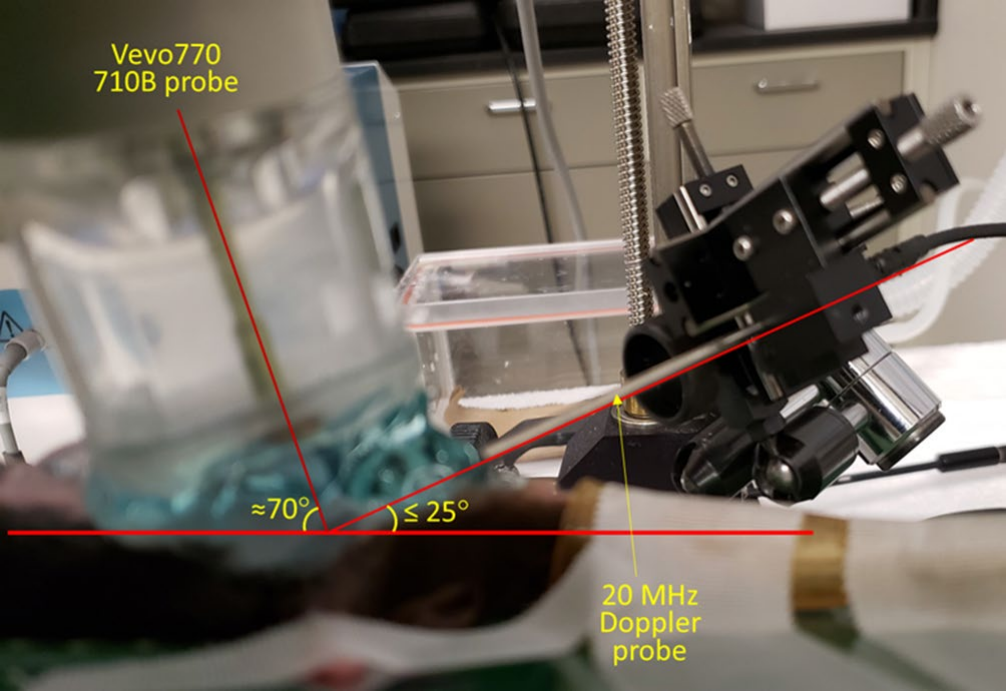
Experimental setup to simultaneously measure ascending aortic Doppler flow velocity using DFVS, left ventricular dimensions and ascending aortic flow velocity using VEVO770 system with 710B probe. Also, shown in the figure are lines representing the horizontal plane, and the angle of orientation of 20 MHz Doppler probe (≤ 25°) and that of the 710B probe (≈70°).

The Doppler (ascending aorta blood flow velocity) measurements and Echocardiography (m-mode and ascending aorta blood flow velocity) measurements were made simultaneously. Aortic blood flow velocity was measured as described in the previous section using the 20-MHz pulsed Doppler probe and DFVS. Again, the Doppler probe was placed slightly to the right of suprasternal notch and oriented towards the heart and aortic root, the probe orientation adjusted with a micromanipulator in the direction from the right ear of the mouse towards the ascending aorta (Fig. 3). In this instance we estimated that the angle between the sound beam and the direction of aortic flow was less than 25° (velocity underestimation error ≤ 9.37%), and the range gate depth was adjusted between 3 and 4 mm to obtain maximal velocity. Echocardiography measurements of m-mode and ascending aorta blood velocity were made with VEVO770 system (VisualSonics, Toronto, ON, Canada) and 710B probe (angle estimated at about 70°—see Fig. 3). Simultaneous measurements were made at baseline and after the IP administration of single dose (1.5 μg/g) of positive inotropic agent dobutamine. From the aortic flow velocity signals measured by both systems peak aortic velocity (v_p_), peak (v′_p_) and mean (v′_m_) acceleration, and peak aortic velocity squared/rise time (v_p_^2^/T) were calculated (as shown in Fig. 2). Also, left ventricular ejection fraction (%EF), fractional shortening (%FS), and cardiac output (CO) were calculated from the dimensions measured by echocardiography. For demonstration of serial Doppler measurements by DFVS only, we used the above data as first day of measurements followed by Doppler measurements made on the fourth and the seventh days.

### Statistics

The body weight of group 1 mice is expressed as mean ± SE (n) and the lowest and highest heart rates are listed. Scatter plots of invasively obtained P′ versus each noninvasively obtained parameters: (1) peak aortic velocity (v_p_), (2) peak aortic velocity squared/rise time (v_p_^2^/T), 3) peak aortic acceleration (v′_p_), and 4) mean aortic acceleration (v′_m_) were made. Linear regression analysis was performed and the respective linear fit relationships, the correlation coefficients (r), and the *p* values were calculated. *p *values < 0.05 were considered significant.

Bland–Altman analysis^26^ was used to evaluate the degree of agreement between P′ and each of the four Doppler derived parameters by plotting the difference against mean of the measurements. The data of all animals were *normalized to unity* (italicized) to compare parameters having different units. The mean of differences (estimation of bias) and standard deviation (measurement error) of: (1) *P*′ vs. *v*_*p*_, (2) *P*′ vs. *v*_*p*_^2^*/T*, (3) *P*′ vs.* v*′_*p*_, and (4) *P*′ vs. *v*′_*m*_, and the respective limits of agreement and 95% confidence intervals were calculated.

The heart rate and body weight of group 2 mice are expressed as mean ± SE (n). The four parameters (v_p_, v_p_^2^/T, v′_p_, and v′_m_) derived from baseline aortic velocity signals measured simultaneously with DFVS and echocardiography were compared. The % change from baseline values of the parameters v_p_, v_p_^2^/T, v′_p_, and v′_m_ obtained by Doppler and echocardiography and the parameters cardiac output, % ejection fraction, and % fractional shortening derived from dimensions measured by echocardiography were calculated and compared. Additionally, the parameters v_p_, v_p_^2^/T, v′_p_, and v′_m_ were calculated from serial repeated Doppler measurements of ascending aortic flow velocity made by DFVS on the first, the fourth, and the seventh day (at baseline and post-dobutamine) and compared using paired t-tests and *p *values < 0.05 were considered significant.

## Results

### Simultaneous invasive pressure and noninvasive Doppler measurements

**Figure 4:**
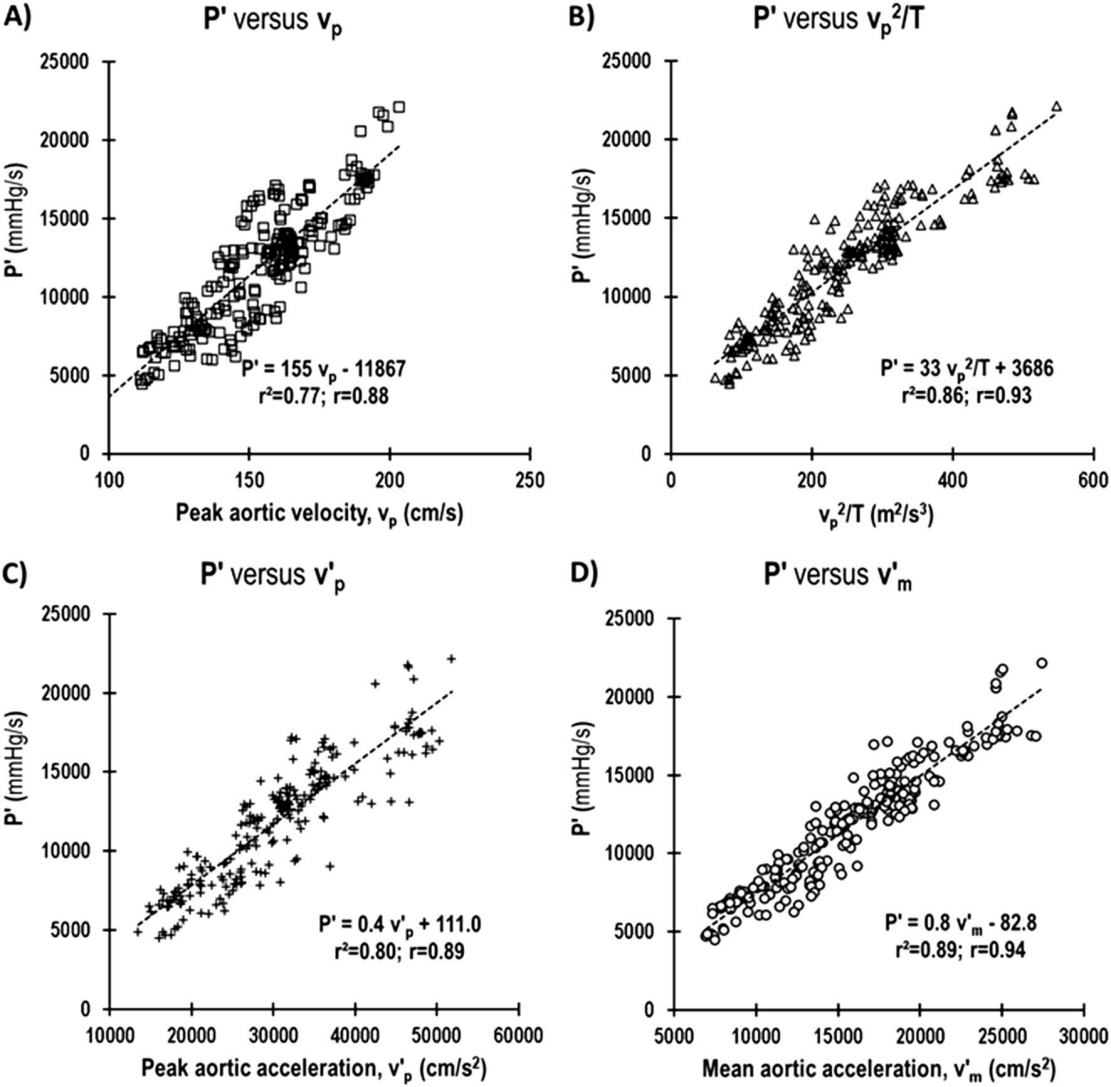
Linear regression analysis of (**A**) peak aortic velocity, v_p_ vs. P′ ( or + dP/dt_max_), (**B**) peak aortic velocity^2^/T, v_p_^2^/T vs. P′, (**C**) peak aortic acceleration, v′_p_ vs. P′, and (**D**) mean aortic acceleration, v′_m_ vs. P′. Shown in each plot is the linear fit equation, the coefficient of determination and the correlation coefficient. The p-values of all were p < 0.0001.

The average body weight (BW) of group 1 mice was 39.0 ± 1.3 g (10) with males weighing more than females. The heart rate ranged from a low heart rate of 387 bpm measured at baseline to a high heart rate of 701 bpm measured after the administration of dobutamine among the 227 data points used for comparison between the methods. The average percentage change in values in response to dobutamine (inotropic effect) was 28% for v_p_, 172% for v_p_^2^/T, 72% for v′_p_, 85% for v′_m_ and 90% for P′. Linear regression analysis of the invasively obtained *P*′ versus each of the four noninvasive Doppler derived parameters (v_p_, v_p_^2^/T, v′_p_, and v′_m_) are shown in Fig. 4. Each plot shows the linear relationship, coefficient of determination (r^2^), correlation coefficient (r), and *p *value for each of the four noninvasive indices.

**Figure 5:**
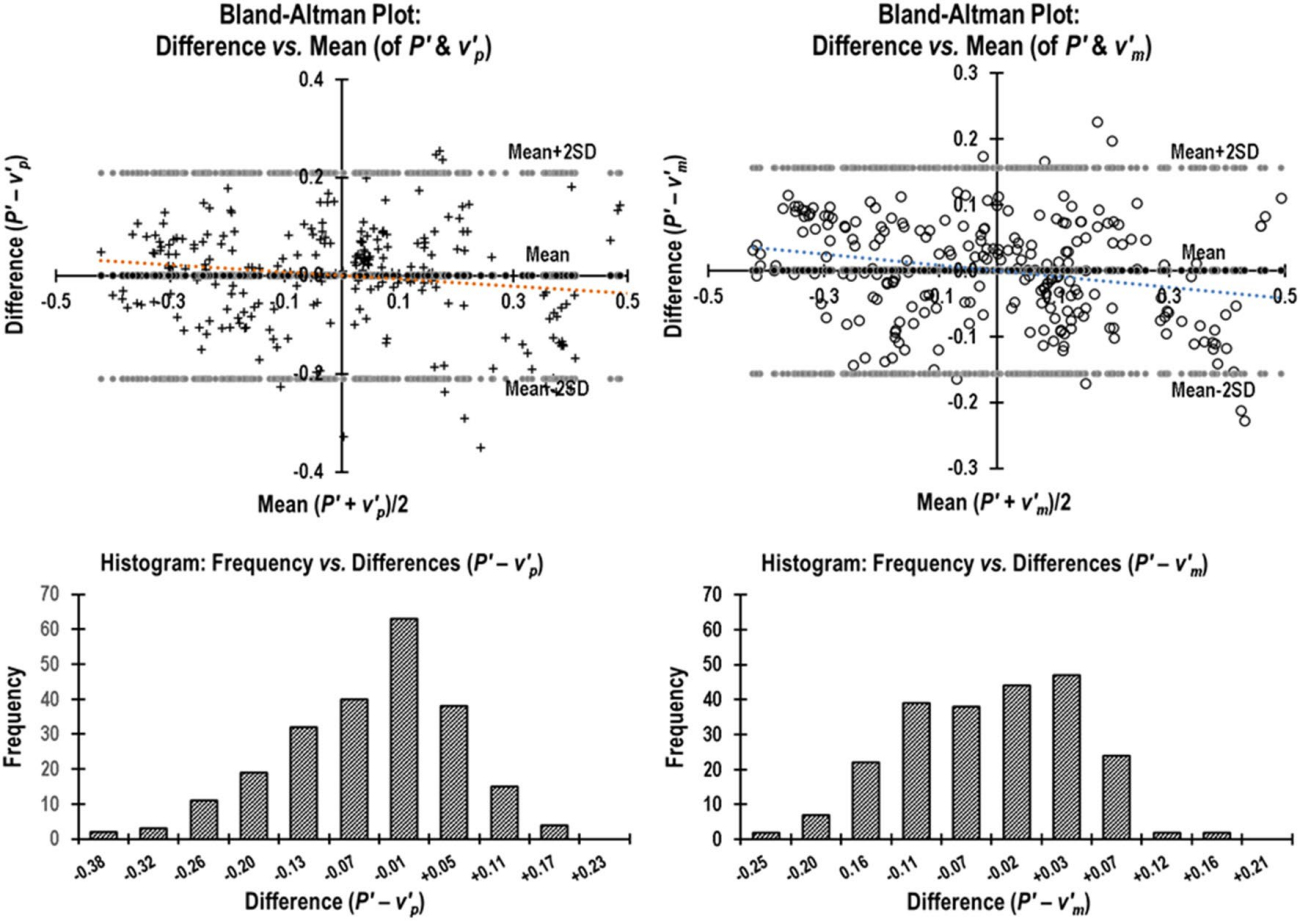
(**A**) Normalized Bland–Altman plot of the agreement between + *dP/dt*_*max*_ (*P*′) and *peak aortic velocity* (*v*_*p*_). Plot of Difference (*P*′–*v*_*p*_) versus Mean ½(*P*′ + *v*_*p*_) along with the histogram distribution of the differences. (**B**) Normalized Bland–Altman plot of the agreement between + *dP/dt*_*max*_ (*P*′) and *peak aortic velocity*^2^*/T* (*v*_*p*_^2^*/T*). Plot of Difference (*P*′–*v*_*p*_^2^*/T*) versus Mean ½ (*P*′ + *v*_*p*_^2^*/T*) along with the histogram distribution of the differences.

**Figure 6:**
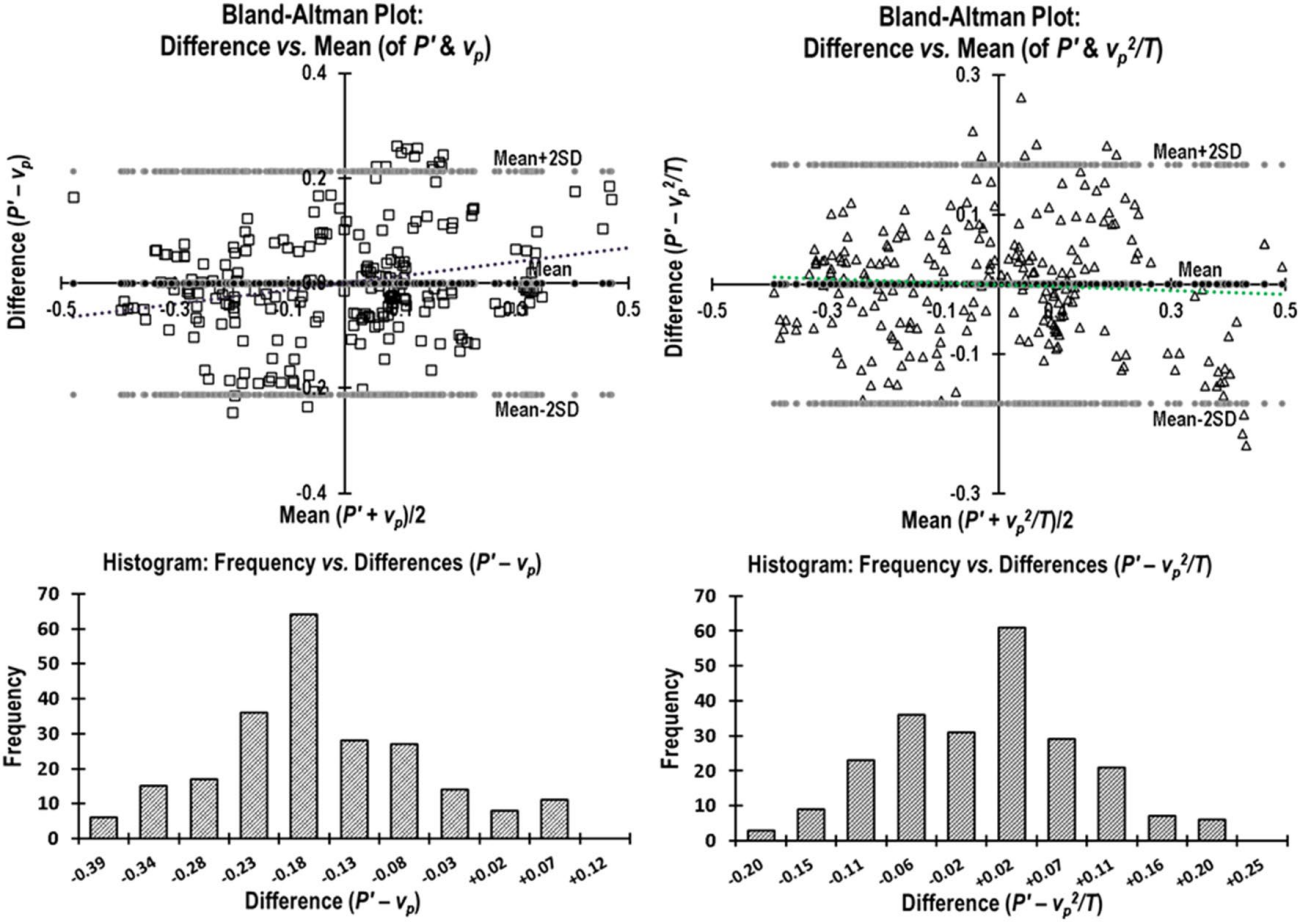
(**A**) Normalized Bland–Altman plot of the agreement between + *dP/dtmax* (*P*′) and *peak aortic acceleration* (v′_*p*_). Plot of Difference (*P*′– *v*′_*p*_) versus Mean ½ (*P*′ + *v*′_*p*_) along with the histogram distribution of the differences. (**B**) Normalized Bland–Altman plot of the agreement between + *dP/dtmax* (*P*′) and *mean aortic acceleration* (*v*′_*m*_). Plot of Difference (*P*′–* v*′_*m*_) versus Mean ½ (*P*′ +* v*′_*m*_) along with the histogram distribution of the differences.

The Bland–Altman analysis plots are shown in Figs. 5 and 6 (*normalized parameters are italicized*). The mean of differences ± SD (mean_diff_ ± SD) and the precision of the estimated limits of agreement (mean_diff_, + 2SD, -2SD) of all measurements based on 95% confidence intervals (CI) are given in Table 1.

**Table 1:** The precision of the estimated limits of agreement obtained from Bland–Altman analysis of all noninvasive measurements compared to the invasively obtained *P*′. Confidence intervals were calculated at 95% for the mean (± CI), upper limit (± CI), and lower limit (± CI) of the differences.

Parameter	*P*′ versus *v*_*p*_	*P*′ versus *v*_*p*_^2^*/T*	*P*′ versus* v*′_*p*_	*P*′ versus *v*′_*m*_
Mean_diff_ (bias)	0.141 ± 0.014	− 0.034 ± 0.011	0.026 ± 0.014	0.018 ± 0.010
Upper limit	0.353 ± 0.024	0.138 ± 0.019	0.235 ± 0.023	0.175 ± 0.017
Lower limit	− 0.072 ± 0.024	− 0.205 ± 0.019	− 0.184 ± 0.023	− 0.138 ± 0.017

### Simultaneous noninvasive Doppler and echocardiography measurements

The average body weight of group 2 mice was 33.7 ± 3.0 g (6) with males weighing more than females. The average heart rate at baseline was 387 ± 27 bpm and post-dobutamine was 516 ± 16 bpm on the first day of serial measurements. The comparison of measurements made with Doppler (DFVS) and echocardiography system only on the first day of serial measurements are shown in Table 2.

**Table 2:** Comparison of the four parameters derived from baseline aortic velocity signals measured simultaneously with DFVS and echocardiography.

Parameter	Doppler No angle correction	Echocardiography with and without Angle correction		t-test Dopp. vs. Echo (0°)	t-test Dopp. vs. Echo (45°)
		None (0°)	45° correction		
v_p_ (cm/s)	90.2 ± 4.4	58.2 ± 8.1	82.3 ± 11.5	*p* < 0.05	*p* = NS
v_p_^2^/T (m^2^/s^3^)	59.3 ± 10.1	27.6 ± 8.5	55.2 ± 16.9	*p* < 0.05	*p* = NS
v′_*p*_ (cm/s^2^)	12,986 ± 1609	3672 ± 880	10,421 ± 2591	*p* < 0.05	*p* = NS
v′_m_ (cm/s^2^)	6449 ± 807	7371 ± 1833	5191 ± 1245	*p* < 0.05	*p* = NS

**Figure 7:**
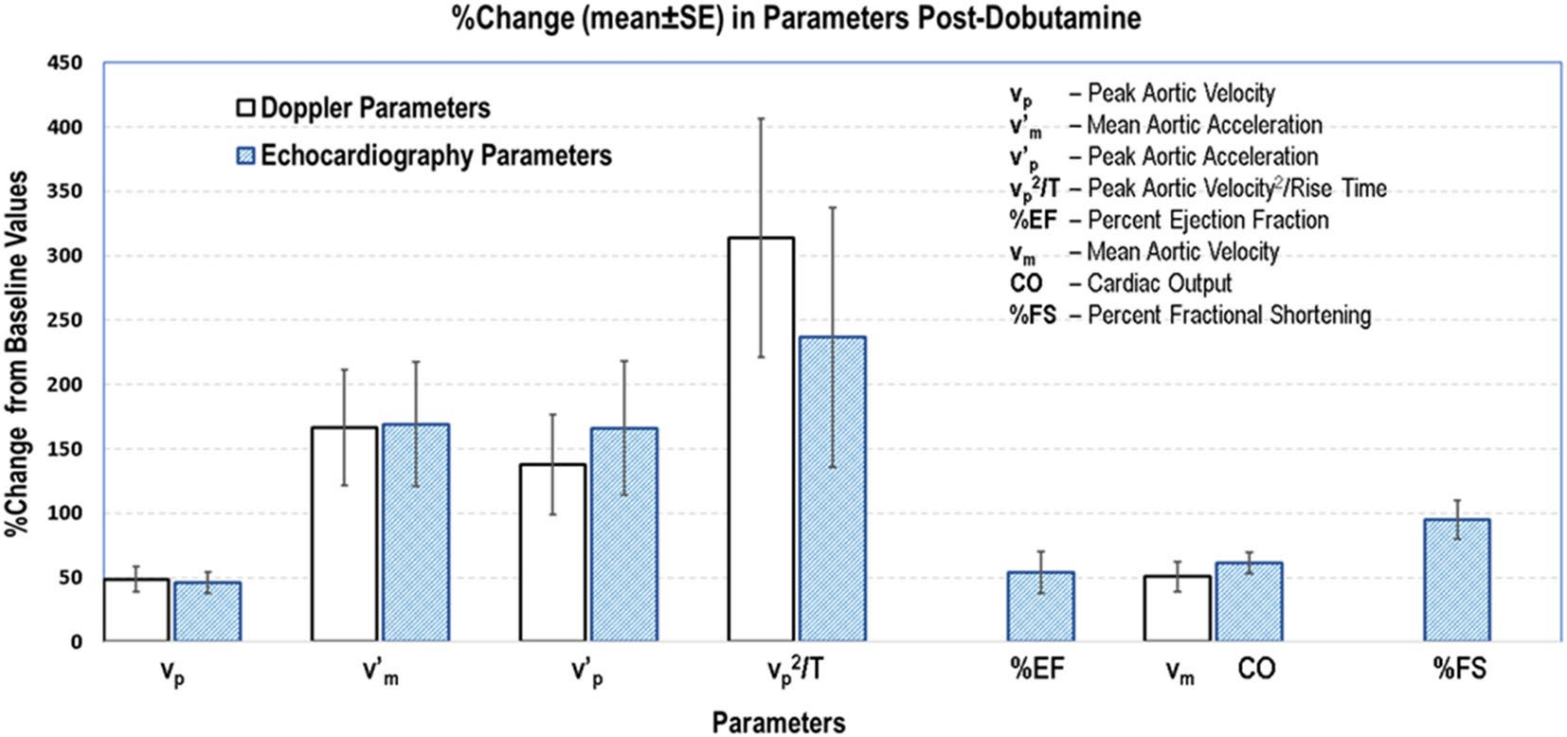
Percent changes in Doppler (DFVS) and Echocardiography (VEVO770) parameters of group 2 mice induced by administration of dobutamine. All dobutamine induced changes were significantly higher regardless of the method used to measure.

From Table 2 we find that without any angle correction the echocardiography measurements are significantly lower than those made with Doppler using DFVS. When we arbitrarily used a 45° angle correction the parameters generated by both methods were not significantly different. Comparison of the % change from baseline values of the parameters v_p_, v_p_^2^/T, v′_p_, and v′_m_ obtained by Doppler and echocardiography and the parameters cardiac output, % ejection fraction, and % fractional shortening derived from dimensions measured by echocardiography showed that all parameters increased significantly from baseline (*p* < 0.05; see Fig. 7).

Table 3 below shows the comparison of parameters body weight, heart rate, v_p_, v_p_^2^/T, v′_p_, and v′_m_ measured by Doppler on first day (D1), fourth day (D4), and seventh day (D7) to demonstrate serial studies. No significant differences were observed in the parameters at baseline of the 3 days. Also, no significant differences were observed in the parameters except between the post-dobutamine heart rates of D1 and D4.

**Table 3:** Serial (repeated) measurement data (body weight, heart rate, v_p_, v_p_^2^/T, v′_p_, and v′_m_) from group 2 mice. Comparison of baseline data (D1 vs. D4; D1 vs. D7; D4 vs. D7) showed no significant differences. Comparison of post-dobutamine data also showed no significant differences except D1and D4 heart rates (*p < 0.05).

Parameters	D1 mean ± SE	D4 mean ± SE	D7 mean ± SE
BW (g)	33.7 ± 3.0	35.7 ± 4.1	35.2 ± 3.7
**Baseline**			
HR (bpm)	387 ± 27	449 ± 20	435 ± 13
v_p_ (cm/s)	90.2 ± 4.4	101.3 ± 6.2	99.4 ± 3.3
v_p_^2^/T (m^2^/s^3^)	59.3 ± 10.1	72.6 ± 21.5	67.8 ± 9.1
v′_p_ (cm/s^2^)	12,986 ± 1609	11,378 ± 2832	14,860 ± 894
v′_m_ (cm/s^2^)	6449 ± 807	7056 ± 624	6820 ± 196
**Post-Dobutamine**			
HR (bpm)	516 ± 16	582 ± 7*	576 ± 16
v_p_ (cm/s)	132.5 ± 3.7	139.0 ± 9.4	140.4 ± 5.1
v_p_^2^/T (m^2^/s^3^)	209.9 ± 22.2	231.0 ± 90.5	234.2 ± 70.3
v′_p_ (cm/s^2^)	28,757 ± 3295	25,629 ± 8000	30,452 ± 3347
v′_m_ (cm/s^2^)	15,804 ± 1380	16,258 ± 1906	16,658 ± 1609

## Discussion

The end systolic pressure–volume relationship is considered as the “gold standard” index of left ventricular contractility but the maximal rate of change of left ventricular pressure (+ dP/dt_max_ or P′) is considered as a reasonable alternative index of contractility^27^. This index has been used in humans^2,28–30^ large animals^7,8,31,32^ and more recently in small animals such as mice^16,18,19,21,22,24,33^. While humans and large animals survive the invasive measurement of left ventricular pressure, in mice this measurement is made acutely and the study is terminal, making longitudinal/serial studies of cardiac contractility/performance impossible. Unlike in large animals, the placement of the catheter in the left ventricle of the mouse forces the ascending aorta and the heart of the mouse to conform to the rigidity of the catheter. This perturbs the mouse hemodynamics to the extent of making the invasive LV contractility measurement in mice less precise. We conducted this study to evaluate the potential use of aortic acceleration obtained from the noninvasively measured aortic blood flow velocity as a surrogate to P′ obtained from the invasively measured LV pressure in mice. To our knowledge this is the first mouse study to perform this evaluation using simultaneously measured LV pressure (and P′) and aortic blood velocity (and v_p_, v_p_^2^/T, v′_p_, and v′_m_).

### Precedence of development of noninvasive indices of cardiac contractility

Ever since the 1960s, researchers have attempted to develop noninvasive indices of cardiac contractility in humans^11,34–36^ and in animal models, like dogs^5,32,37,38^, pigs^7,39^, sheep^8,40^, rabbits^41^, rats^9,42,43^ and even in mice^44^. The noninvasive methods included echocardiography^44–46^, tissue Doppler imaging^47^, Tei index^48,49^, maximal rate of change aortic volume flow^38^, to list a few. The imaging methods are cumbersome and time consuming; the tissue Doppler imaging and Tei index calculation depend on the quality of the acquired signals and time resolution, and volume flow measurements are extravascular and partially invasive.

### Justification for aortic flow velocity parameters as indices of cardiac contractility

The results from our study demonstrate that there is significant correlation between the invasively derived maximal rate of change of left ventricular pressure (P′) and each of the noninvasively derived indices of: peak aortic velocity (v_p_), peak aortic velocity squared/rise time(v_p_^2^/T), peak aortic acceleration (v′_p_), mean aortic acceleration (v′_m_).

The justification for use of peak aortic flow velocity (v_p_) to define ventricular contractility comes from the definition by Rushmer^31^ that the initial ventricular impulse is the net force acting over the time from the beginning of ejection to the attainment of peak aortic flow velocity. This net force is due the initial rapid increase in LV pressure as determined by its rate of change and the rate of blood flowing into the aorta during the initial phase of ejection^31^. We found that P′ was well correlated to v_p_ (r = 0.88, Fig. 4A) and in agreement with the findings by others^11,34,35^, but found that correlation coefficient of P′ versus v_p_ was the lowest of the four parameters we evaluated. Hunt et al. also found that P′ had the lowest correlation to v_p_^34^ and Lambert et al. reported that that v_p_ was much less sensitive than mean or peak aortic accelerations in assessing LV contractility^25^.

The parameter peak aortic velocity squared/rise time (v_p_^2^/T) was adopted from Hunt et al., who derived a mathematical equation from the basic principle that force generated by LV translates into kinetic energy of blood flow, implying that P′ was directly proportional to v_p_^2^/T and found that v_p_^2^/T had the best correlation with P′ in humans (r = 0.77, *p* < 0.001)^34^. In this study we found P′ had the second highest and significant correlation v_p_^2^/T (r = 0.93; Fig. 4B).

The force generated by the contracting ventricle causes the acceleration of blood into the aorta. The maximum generated force to overcome inertia in early systole results in peak aortic blood acceleration (v′_p_), which decreases rapidly before any changes occur, and is therefore considered as the most sensitive index of myocardium contractility^37^. In our study v′_p_ was also highly correlated P′ (r = 0.89; Fig. 4C) and agrees with the findings by others^29,37,50^. According to Lambert et al. peak aortic acceleration is the second most sensitive of all the noninvasive parameters^25^. Harada et al. showed that the product of v′_p_, pulse wave velocity (c), and the density of blood (ρ) highly correlated with P′ (ρc⋅ v′_p_ = 1.01P′−2, r = 0.97)^5^. We were unable to confirm this as we did not measure pulse wave velocity in this study. Pulse wave velocity measurement requires the placement of Doppler probes at two additional arterial sites and this would increase the amount of time required for each LV contractility measurement.

Aortic velocity waveforms are computed from Doppler spectrograms which can be noisy. Determination of peak aortic acceleration (v′_p_) requires high fidelity aortic velocity waveforms that need to be sampled at high rates such that a less noisy first derivative can be calculated. So, mean aortic acceleration (v′_m_ = v_p_/T), which is simpler to calculate is used instead of peak aortic acceleration. Of the four indices we evaluated, we found that v′_m_ had the highest correlation with P′ (r = 0.94; Fig. 4D). In humans^34^ and dogs^38,50^ v′_m_ was found to be significantly correlated to P′. Lambert et al. found v′_m_ to be the most sensitive of all the noninvasive parameters^25^. Bauer et al. reported that mean aortic acceleration had a strong correlation with maximal LV elastance (another contractility index)^8^.

Since we compared two parameters whose units were different, we *normalized* each data set to unity (like that reported by Morimont et al.^27^) to perform Bland–Altman tests. Bland–Altman analysis plots (Fig. 5 and 6) revealed good agreement between the invasive obtained *P*′ and each of the noninvasively derived parameters *v*_*p*_, *v*_*p*_^2^*/T*, v′_*p*_, and v′_*m*_. From Table 1, we can see that the 95% CI of estimated limits of agreement are quite narrow and, therefore, acceptable. We cannot compare our findings from Bland–Altman analysis of our parameters with other studies as those studies have not performed the Bland–Altman analysis.

### Comparison to echocardiography parameters

Comparison of the four aortic flow velocity parameters without using angle correction showed that the echocardiography method significantly underestimated these parameters (Table 2). When a 45° angle correction was applied only to the signal measured by echocardiography the differences were not significant, but the values were still lower than those measured by DFVS. We did not apply an angle correction (of ≤ 25°) to the Doppler measurements and this would result in a < 10% error in estimating peak aortic velocity. The results we obtained with echocardiography in estimating peak aortic velocity and accelerations agree with the finding by Yang et al.^51^ that the VEVO770 with 710B probe results in measurement error of about 35% at 40° and about 82% at 70°. In our study the measurements made by echocardiography underestimated peak aortic velocity by 35.5% without angle correction and by 8.3% with a 45° angle correction when compared to the measurement made by DFVS. Other investigators have suggested that for a probe orientation at above 60° the smallest error in estimating the true angle can result in large measurement errors^52^. We, however, believe that the measurement error needs to be kept as small as possible if serial measurements are planned. With the small footprint of our Doppler probes it is possible for us to achieve angles as low as 10° between the ultrasound beam and the axial direction of blood flow. We previously reported that measurement of Doppler velocity with angle correction within 5° of the true angle, if the probe angle is 25°or less, limits the measurement error to less than 5%^53^. A noteworthy observation is that, when baseline-to-post-dobutamine %changes were considered, both methods yielded similar results regardless of angle correction.

The percent changes in baseline-to-post-dobutamine responses also showed that all aortic velocity parameters (by both Doppler & echocardiography) including mean aortic velocity (v_m_; by Doppler) increased significantly in the same direction as cardiac output, % ejection fraction, and % fractional shortening derived from LV dimensions measured by echocardiography (Fig. 7). In the case of myocardial infarction measurements of LV dimensions with echocardiography may depend on where the probe is placed, that is, measurements made in non-infarcted zone can produce results that are totally different from the measurements made from infarcted zone or from somewhere in between the two zones. Serial assessment of function also depends on the consistency of the operator making the measurements at the same location at each time point. By contrast, peak aortic flow velocity and its acceleration represent a comprehensive global systolic (contractile) function with little ambiguity.

### Repeatability studies

The body weights, heart rates, and the four aortic flow velocity parameters (v_p_, v_p_^2^/T, v′_p_, and v′_m_) measured in the same group 2 mice on 3 different days showed no significant differences in baseline measurements indicating that serial studies done within a short period of time in normal animals do not show significant changes within a few days thereby demonstrating repeatability. In a previous study our group has shown that peak aortic velocity (v_p_) obtained from repeated measurements of aortic flow velocity can be used to study systolic function in mouse models of myocardial remodeling^54^. Serial measurements of v_p_ were made in 3 groups of mice categorized as sham controls (SC), permanent occluded (PO), and 2-h occlusion/reperfusion (OR) (of left anterior descending coronary artery) over a period of up to 6 months of age. They reported that v_p_ in both PO and OR groups dropped to about 75% of baseline values, followed by a recovery in the OR group to the same levels as SC by the 8th week, but the PO group remained at about 75% of the baseline at about 6 months indicating a significant decrease in systolic function.

### Advantages and potential applications

**Figure 8:**
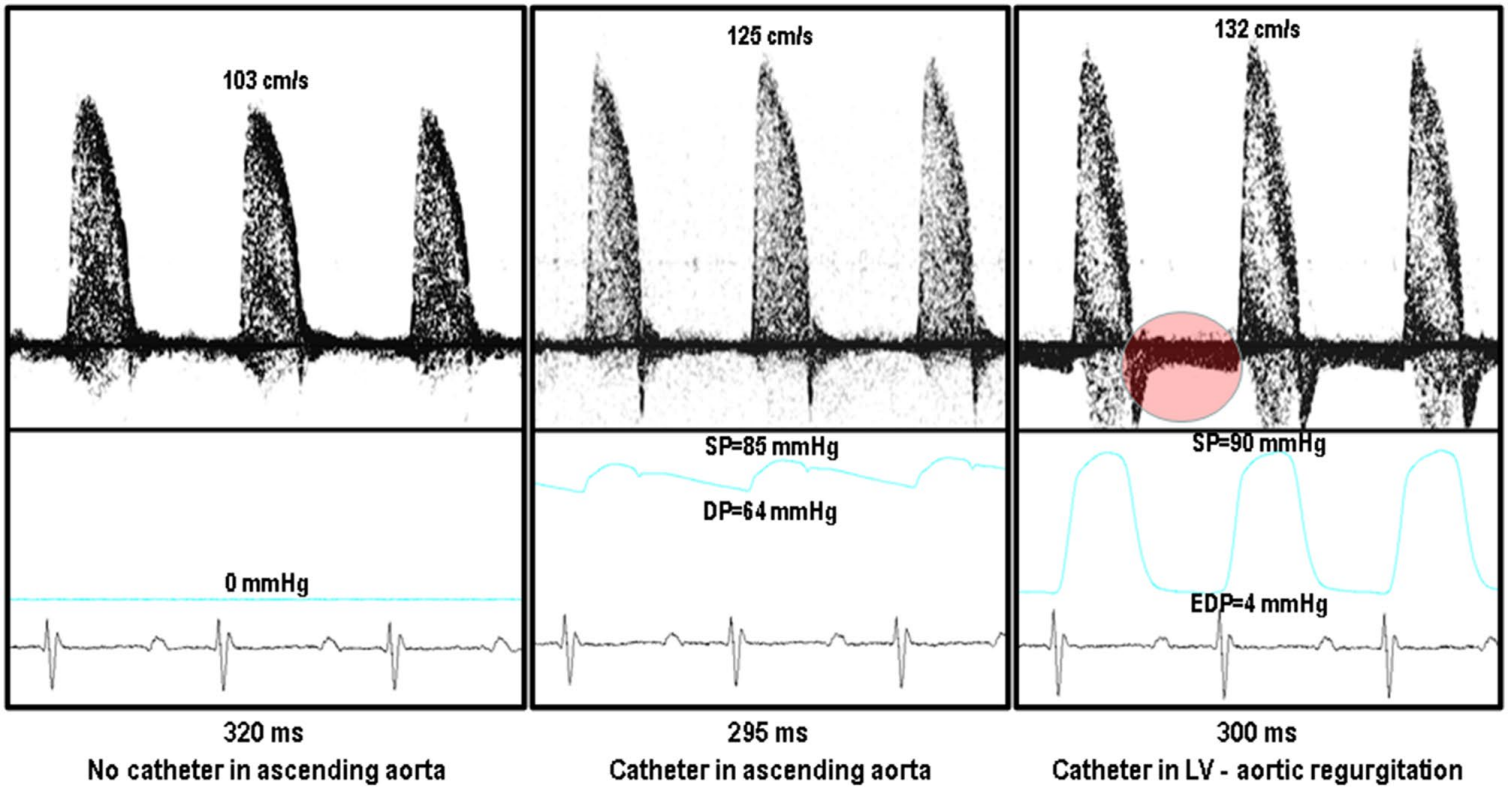
Ascending aortic flow velocity at (**A**) no intravascular pressure catheter, (**B**) intravascular pressure catheter in the ascending aorta, and (**C**) intravascular pressure catheter in the left ventricle via aortic valves (presence of aortic regurgitation—see circle).

One obvious advantage of noninvasive measurements is that they can be made repeatedly over the short-term or long-term serially using an animal as its own control. The second advantage is that these measurements can be done without significantly altering the physiology. Shown in Fig. 8 are three conditions under which aortic flow velocity signals are measured in the same mouse. The first panel shows an aortic flow velocity spectrogram in an intact animal with v_p_ around 100 cm/s (considered normal). The second panel shows the simultaneous measurement of aortic velocity spectrogram (with v_p_ increased to 125 cm/s) and aortic pressure signal measured with a 1F (0.33 mm diameter) intravascular catheter. The third panel shows the simultaneous measurement of aortic velocity spectrogram and LV pressure signal (with the same catheter across the aortic valve). In this case v_p_ is further increased to 132 cm/s along with some aortic regurgitation.

Using an allometric equation, AD = 3.6 BW^3/8^, relating aortic diameter (AD) to body weight (BW) in mammals^55^ we estimated that the aortic diameter in this 34 g (0.034 kg) mouse was 1.01 mm resulting in a luminal area of 0.8012 mm^2^. Thus, the presence of the catheter (0.33 mm dia. or 0.0855 mm^2^ cross-sectional area) in the lumen will cause about 12% increase in v_m_ and peak aortic velocity is expected to change in a similar way. In this mouse we found that v_p_ increased by 21% with the catheter in the aorta and increased by another 7% when the catheter was advanced into the LV. The presence of aortic regurgitation calls into question the correction made to the P′ using LV end diastolic volume or pressure to account for preload.

The validation and use of aortic acceleration as an index of cardiac contractility in humans and large animals has suggested its utility in mice, despite not being evaluated. Our study suggests that this technique is useful in allowing longitudinal studies in mice to assess changes with age or interventions. Previously our group has reported the use of mean aortic acceleration to compare cardiac function in mice that have been injected with saline or AICAR^56^. or to describe cardiac function in desmin null mice with Bcl-2 overexpression^57^, or to study the effects of TAC in SRC-2 KO mice^58^. Others have reported the use of both peak and mean aortic acceleration to describe cardiac function at baseline and post-dobutamine in ApoE-KO mice^59^. The measurements of aortic velocity signal in all these studies were made with a high-speed and high-fidelity Doppler system (DFVS) and signals were acquired at high sampling rates. The peak velocity envelope calculated by the analysis software^60^ is critical in calculating the Doppler indices. Another advantage is that Doppler measurements can be made much faster with DFVS than with echocardiography.

### Limitations of the study

LV dP/dt_max_ (P′) can be affected by preload, afterload, heart rate and myocardial hypertrophy. In this study we did not make measurements with changes to preload or afterload. Under the conditions of this study we assumed that preload and afterload are unchanged. Since we are studying the relative responses of invasive and noninvasive parameters to changes in hemodynamics, we assumed that preload and afterload are not significantly altered with single administration of dobutamine. As Lambert et al. have alluded to, experimentally induced changes to preload and afterload are less physiological and therefore, may be less relevant in this evaluative study^25^. Wallmeyer et al. noted that the correlation between the Doppler indices and P′ was not significantly affected after adjusting preload or heart rate and concluded that both noninvasive and invasive indices are similarly affected by the above variables^38^.

A limitation of noninvasive Doppler indices is that they do not directly measure cardiac contractility^34,38^ and therefore are only as sensitive as invasive indices. Since the noninvasive indices do not describe the inotropic state in absolute terms they can be only used for comparisons within a subject (that is, each subject as its own control) which is more practical in animal studies than in clinical applications.

Echocardiography was used in several studies^8,44–46^ to measure aortic flow velocity, but we believe that echocardiography measurement of aortic outflow in mice may not be sensitive enough for serial measurements of LV contractility. Bauer et al. however, noted that v′_m_ calculated from aortic velocity measured at LV outflow tract (LVOT) in sheep consistently reflected changes observed in LV contractility^8^. All our measurements of aortic velocity were made in the LVOT using a 2 mm Doppler probe mounted on a micromanipulator to optimize the signal and stabilize the position.

### Future studies

We did not evaluate the noninvasive indices with the administration of a negative inotrope, which would have provided a reasonable representation of heart failure models. In our future studies we will include negative inotrope intervention as well as preload and afterload variations and study the effects on Doppler indexes of left ventricular performance, and to help identify the Doppler index that best reflects changes in contractility. Since this study was done using young mice, we will conduct studies to validate these results in middle-aged and old mice.

## Conclusions

We compared four parameters (peak aortic velocity, peak aortic velocity squared/rise time, peak aortic acceleration, and mean aortic acceleration) derived from aortic velocity waveform to the first derivative of LV pressure. The aortic velocity and the LV pressure waveforms were measured simultaneously at baseline and after the administration of dobutamine. The results showed that the four noninvasive indices had a high degree of correlation and agreement with the first derivative of LV pressure, indicating that mean or peak aortic acceleration, or peak aortic velocity squared/rise time or even peak velocity derived from aortic velocity may be used as a noninvasive index of LV contractility. We also performed repeatability studies to successfully demonstrate that noninvasive serial studies can be done with ease.

## Data Availability

The datasets generated during and/or analyzed during the current study are available from the corresponding author on reasonable request.
