# Author Correction: Aortic acceleration as a noninvasive index of left ventricular contractility in the mouse

**DOI:** 10.1038/s41598-021-87101-5

**Published:** 2021-04-06

**Authors:** Jorge Enrique Tovar Perez, Jesus Ortiz-Urbina, Celia Pena Heredia, Thuy T. Pham, Sridhar Madala, Craig J. Hartley, Mark L. Entman, George E. Taffet, Anilkumar K. Reddy

**Affiliations:** 1 Section of Cardiovascular Research, Department of Medicine, Baylor College of Medicine, One Baylor Plaza, MS:BCM285, Houston, TX 77030 USA; 2 Houston Methodist Hospital, Houston, TX USA; 3 Texas A&M University, Houston, TX USA; 4 Indus Instruments, Webster, TX USA

Correction to: *Scientific Reports* 10.1038/s41598-020-79866-y, published online 12 January 2021

In the original version of this Article, the author Jorge Enrique Tovar Perez was incorrectly indexed.

In addition, Table 2 contained an error in the value for ‘v′_*p*_ (cm/s^2^)’ in ‘Doppler No angle correction’. The original Table 2 and accompanying legend appear below.

**Table 2:** Comparison of the four parameters derived from baseline aortic velocity signals measured simultaneously with DFVS and echocardiography.

Parameter	Doppler No angle correction	Echocardiography with and without Angle correction		t-test Dopp. vs. Echo (0°)	t-test Dopp. vs. Echo (45°)
		None (0°)	45° correction		
v_p_ (cm/s)	90.2 ± 4.4	58.2 ± 8.1	82.3 ± 11.5	*p* < 0.05	*p* = NS
v_p_^2^/T (m^2^/s^3^)	59.3 ± 10.1	27.6 ± 8.5	55.2 ± 16.9	*p* < 0.05	*p* = NS
v′_p_ (cm/s^2^)	6449 ± 1609	3672 ± 880	5191 ± 1245	*p* < 0.05	*p* = NS
v′_m_ (cm/s^2^)	12,986 ± 1609	7371 ± 1609	10,421 ± 2591	*p* < 0.05	*p* = NS

The original Article has been corrected.

