# Author Correction: Aortic acceleration as a noninvasive index of left ventricular contractility in the mouse

**DOI:** 10.1038/s41598-021-03203-0

**Published:** 2021-12-03

**Authors:** Jorge Enrique Tovar Perez, Jesus Ortiz-Urbina, Celia Pena Heredia, Thuy T. Pham, Sridhar Madala, Craig J. Hartley, Mark L. Entman, George E. Taffet, Anilkumar K. Reddy

**Affiliations:** 1 Section of Cardiovascular Research, Department of Medicine, Baylor College of Medicine, One Baylor Plaza, MS:BCM285, Houston, TX 77030 USA; 2 Houston Methodist Hospital, Houston, TX USA; 3 Texas A&M University, Houston, TX USA; 4 Indus Instruments, Webster, TX USA

Correction to: *Scientific Reports* 10.1038/s41598-020-79866-y, published online 12 January 2021

The original version of this Article contained errors in Table 2, where the values for echocardiography category were incorrect for “v’_*p*_ (cm/s^2^)” and “v’_m_ (cm/s^2^)”. The correct and incorrect values appear below.

Incorrect:

**Table:** 

Parameter	Doppler No angle correction	Echocardiography with and without Angle correction		t-test Dopp. vs. Echo (0°)	t-test Dopp. vs. Echo (45°)
		None (0°)	45° correction		
v’_*p*_ (cm/s^2^)	6449 ± 807	3672 ± 880	5191 ± 1245	*p* < 0.05	*p* = NS
v’_m_ (cm/s^2^)	12,986 ± 1609	7371 ± 1609	10,421 ± 2591	*p* < 0.05	*p* = NS

Correct:

**Table:** 

Parameter	Doppler No angle correction	Echocardiography with and without Angle correction		t-test Dopp. vs. Echo (0°)	t-test Dopp. vs. Echo (45°)
		None (0°)	45° correction		
v’_*p*_ (cm/s^2^)	12,986 ± 1609	3672 ± 880	10,421 ± 2591	*p* < 0.05	*p* = NS
v’_m_ (cm/s^2^)	6449 ± 807	7371 ± 1833	5191 ± 1245	*p* < 0.05	*p* = NS

